# Why Girls Go Wrong

**Published:** 1913-08

**Authors:** 


### Why Girls Go Wrong.

"The family is the unit of civilization; if the family life is wholesome and pure, the community life reflects these attributes and becomes pure and wholesome, but if the family life is bad and governed by low ideals or none, the community life at once becomes depraved and vicious. Here then we have the origin of crime and lawlessness in all of its different phases and the starting point on the road that leads to a life of shame and disgrace. The parents in the family circle are supposed to instill into the minds of their children obedience to law, high ideals, strictly moral sentiments, all of which lead to a high grade citizenship. Without these lessons in the home, how can we expect the rising generation to become honored and respected citizens? Who will expect them to be obedient to the laws of the land, when obedience had not been taught and demanded at home? Want of the exercise of parental control and discipline in the household has furnished more dwellers in the red-light district than all other causes combined, and compared with which the cause of low wages sinks into utter insignificance. Want of parental control leads the young and inexperienced girl into the streets of our cities and towns, into the low dance halls, into the companionship of unprincipled men who take advantage of her inexperience, and she soon falls a victim to their evil wiles. We see scores and hundreds of young girls unattended roaming our streets at all hours of the night, going to picnics, balls and summer gardens unchaperoned, and then we wonder why it is that new candidates for the haunts of vice are constantly on hand. The truth of the matter is that in the great majority of the homes of this land every vestige of discipline has disappeared, and that girls even at the tender age of 12 to 14 years go where they please and do as they please, without any regard to the wishes of the parents. This, as we see it, is the great cause of the social evil, overshadowing all other causes. An editorial in the *Edwardsville Intelligencer* is right to the point when it says:

"Why do girls go wrong—because men are depraved. Because the fathers do not instill in the minds of their sons a manly respect for womanhood,—nor for the desirability of that portion of masculine honor which includes self-respect. Because the mothers who would carefully direct a daughter how to find her way about an unknown city never prepares the younger mind for the pathway which leads through the unchaperoned dance, the lonely buggy ride, the moonlight stroll.

We don't tell either the girl or the boy the difference between good and vile as concerns relations of the sexes, but let them drift. Some girls go wrong because they are naturally and temperamentally impressible. Very few indeed, probably not one in a thousand, because her wages are too low. Just as many girls fall who are in comfortable homes and who do not know the need of employment as those who work for their living. Idleness, in fact, is far more dangerous than employment. If the vice commission wants to really trace the evils, let them visit the "family entrance" resorts, the garish dance halls, and the ribald rinks. Let them get some statistics on how many parents ever inquire into the qualities of the young men who seek the company of their daughters. Let them pursue the subject even into the sacred precincts of "high society," with its vulgar and suggestive dances. Let them wander to the public schools and learn some of the language and thoughts that other boys on the playgrounds put into the minds of our own boys."

The *Edwardsville Democrat* is on the right track, too. "It is injustice to charge more than a small per cent of the cause to department stores, factories, etc. Keep the girls away from Sunday night picnics and dances, park strolls and midnight orgies, and a large portion of the downfall of the younger girls will be thus avoided. In hunting for reasons, don't grab at the wrong ones."

There you have it in a nutshell. If every family would take care of its own, if every mother would guide the faltering footsteps of her daughter through the perilous years of inexperienced youth, there would be no need of a vice commission; there would be no underworld, there would not be so many heartbreaks nor so many years of frightful agony. Make the home life attractive so that your daughter would rather be at home than elsewhere. Surround her with honorable, respectable companions of her own age and of either sex. But whether the home is attractive or not, it is a far safer place for her than the music hall with its "bunny hug" and "turkey trot," the moonlight walk, or ride, in the company of some disreputable man. Guard the home.—*The Madison County Doctor.*

